# Revision Anterior Cruciate Ligament Reconstruction Using Rectus Femoris or Hamstring Tendon Shows Comparable Patient-Reported Outcome Measurements at Short-Term Follow-Up: A Retrospective Cohort Study

**DOI:** 10.3390/jcm14103512

**Published:** 2025-05-17

**Authors:** Thorsten Huber, Marcel Frühwirth, Florian Hartenbach, Sarah Franzmair, David Ullmann, Klemens Trieb, Björn Rath

**Affiliations:** 1 Department of Orthopedics, Klinikum Wels-Grieskirchen, 4600 Wels, Austria; thorsten.huber@klinikum-wegr.at (T.H.); sarah.franzmair@klinikum-wegr.at (S.F.); david.ullmann@klinikum-wegr.at (D.U.); bjoern.rath@klinikum-wegr.at (B.R.); 2 Department of Trauma Surgery, Klinikum Wels-Grieskirchen, 4600 Wels, Austria; florian.hartenbach@klinikum-wegr.at; 3 Department of Orthopedics, Paracelsus Medical University, 5020 Salzburg, Austria; k.trieb@salk.at; 4 Division for Orthopaedics and Traumatology, Center for Clinical Medicine, Danube Private University, 3500 Krems, Austria

**Keywords:** rectus femoris tendon graft, ACL autograft, revision anterior cruciate ligament reconstruction

## Abstract

**Background:** The isolated rectus femoris tendon (RT) is a less commonly used autograft for anterior cruciate ligament (ACL) reconstruction. Graft selection is a critical part of ACL reconstruction, especially in revision surgery. Hypothesis: This study compares patient-reported outcome measurements (PROMs) between revision ACL reconstruction with an RT autograft and a hamstring tendon (HT) autograft. We hypothesized that the RT autograft will yield comparable functional results and high patient satisfaction. Study Design: This was a cohort study; the level of evidence is III. **Methods:** Fifty-five patients (RT n = 28; HT n = 27) who underwent revision ACL reconstruction were included in this study, with a mean follow-up time of 40.3 months (range, 16.4–64.8) for RT and 61.2 months (range, 34.6–86.3) for HT. Apart from the harvesting technique, the surgical technique was the same for both groups. Clinical and intraoperative data were collected for our postoperative registry. In addition, funcinal outcome was measured using the International Knee Documentation Committee score (IKDC), the Lysholm score, Tegner activity scale, and numeric rating scale (NRS). The type and frequency of postoperative complications were documented. **Results:** At the final follow-up, no significant differences were observed between the RT and HT groups in the IKDC (mean ± SD: 74.7 ± 10.9 vs. 74.9 ± 12.9), Lysholm score (90.9 ± 15.0 vs. 89.0 ± 14.6), or Tegner activity scale (median [IQR]: 5 [4–6] vs. 5 [4–6]). The mean femoral tunnel diameter was 9.0 mm (range, 7.5–10 mm) for the RT and 8.2 mm (range 7.0–9.5 mm) for the HT. The use of the RT reduced the need for a two-stage procedure by 50% compared to HT (n = 5 vs. n = 10), although this difference was not statistically significant (*p* = 0.11). Stability measured by the Lachman test improved significantly in both groups from the pre- to postoperative period. Retear of the ACL graft was observed in two patients in both groups (7.1% RT and 7.4% HT). **Conclusions:** The RT and HT autografts achieved similar outcomes in PROMs for revision ACL reconstruction. Good tendon quality with parallel fibers and adjustable thickness characterize the RT. A tendency for a reduced rate of two-stage surgery with RT was noticed. Clinical Relevance: The incidence of revision ACL reconstruction is rising. Surgeons should be aware of all the available graft options. The isolated RT expands the range of autografts available for ACL reconstruction.

## 1. Introduction

Various factors determine the success of ACL reconstruction. An essential part of surgical and postoperative management is graft selection. Graft selection is crucial for the stability and functional outcome of the injured knee joint [1,2,3]. In revision ACL surgery, the graft selection depends not only on the surgeon’s preference but also on factors such as the previously used tendon or bone tunnel enlargement.

Hamstring tendon (HT) autografts are widely used in anterior cruciate ligament reconstruction. However, limitations have been noted regarding the initial fixation strength, with risks of graft micromotion and prolonged integration within the bone tunnel [4]. Furthermore, the adaptation of tendon thickness in the case of tunnel enlargement is limited using this graft.

In recent years, the quadriceps tendon (QT) has become increasingly popular for ACL reconstruction. Many studies have shown good functional outcomes, high stability, and low harvest morbidity [3,5,6,7,8,9,10,11,12]. Critically, the QT is composed of several parts and fibers with different directions of traction [13]. In line with most surgical methods, a graft is created from all parts of the QT [11,14]. The longitudinal parts of the QT come from the rectus femoris and the vastus intermedius. The tendon segments of the vastus lateralis and vastus medialis muscles are said to contribute to the volume of the graft, but they do not enhance its strength. Consequently, this may lead to fiber ruptures and the elongation of the graft [15,16,17]. So far, there are only a few studies and technical notes that have described the rectus femoris tendon (RT), the superficial part of the QT, for ACL reconstruction [18,19,20,21].

Our study aims to compare the patient-reported outcome measurements (PROMs) of patients undergoing revision ACL reconstruction using an RT autograft and an HT autograft. We hypothesized that the RT autograft will lead to similar postoperative functional outcomes and high patient satisfaction. Comparable outcomes were anticipated for the International Knee Documentation Committee (IKDC) score, Lysholm score, Tegner activity level, and numeric rating scale (NRS). Additionally, we expected that the rate of two-stage surgical procedures will be reduced with the RT due to the adaptable tendon thickness achievable with our technique.

## 2. Materials and Methods

This study was conducted and reported following the STROBE (Strengthening the Reporting of Observational Studies in Epidemiology) guidelines [22]. It was performed as a retrospective, case–control study with a proposed final follow-up investigation and the implementation of PROMs.

We used our postoperative registry to identify patients who underwent revision ACL reconstruction using an RT autograft. All 38 cases between March 2017 and May 2021 were screened for eligibility. A total of 28 patients wanted to participate in the follow-up, met the inclusion criteria, and were included in our series. This cohort was matched with respect to individuals who underwent ACL revision surgery, who were treated with a single-bundle HT autograft. Informed consent was obtained from each patient. The inclusion criteria were revision ACL reconstruction, ligament reconstruction with an RT or HT autograft, no specific gender distribution, and a minimum follow-up of 12 months. The exclusion criteria were open epiphyseal plate, >3 previous knee surgeries, morbid obesity (Body Mass Index > 40 kg/m^2^), skeletal malformation, intraarticular infection, and arthrofibrosis. Most patients included in this cohort were recreational or semi-professional athletes who sustained ACL injuries during sports activities. Pre-injury activity levels were assessed via the Tegner scale.

Wherever available, information regarding the technique used in the initial ACL reconstruction was extracted from patient records. Most initial reconstructions were single-bundle procedures using the ipsilateral hamstring tendon

This study was approved and registered by the local ethics committee and conducted according to the principles expressed in the Declaration of Helsinki (EK Nr: 1106/2021). Written informed consent was obtained from all patients for participation and data usage.

### 2.1. Surgical Technique

All patients in the RT group were treated by one experienced knee surgeon (second author’s name), who began using this technique in March 2017. Apart from the harvesting technique, the surgical technique was the same for both groups.

### 2.2. Harvest of RT

**Figure 1:**
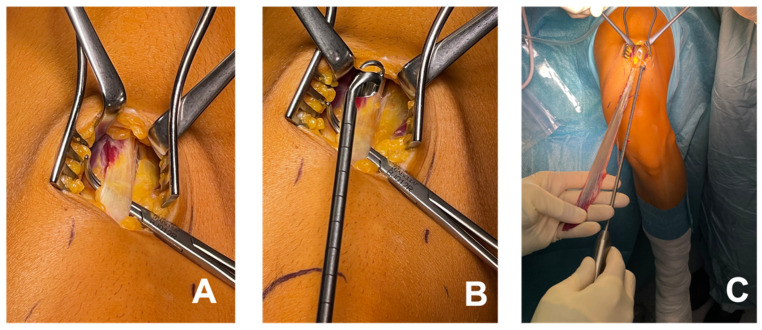
An illustration of rectus tendon harvesting. An incision measuring 3 cm is created near the proximal patellar pole to expose the tendon (**A**). Using an open stripper, the tendon is detached proximally (**B**,**C**). The length of the harvested rectus femoris tendon was measured intraoperatively. On average, a graft length of 30–34 mm was obtained.

**Figure 2:**
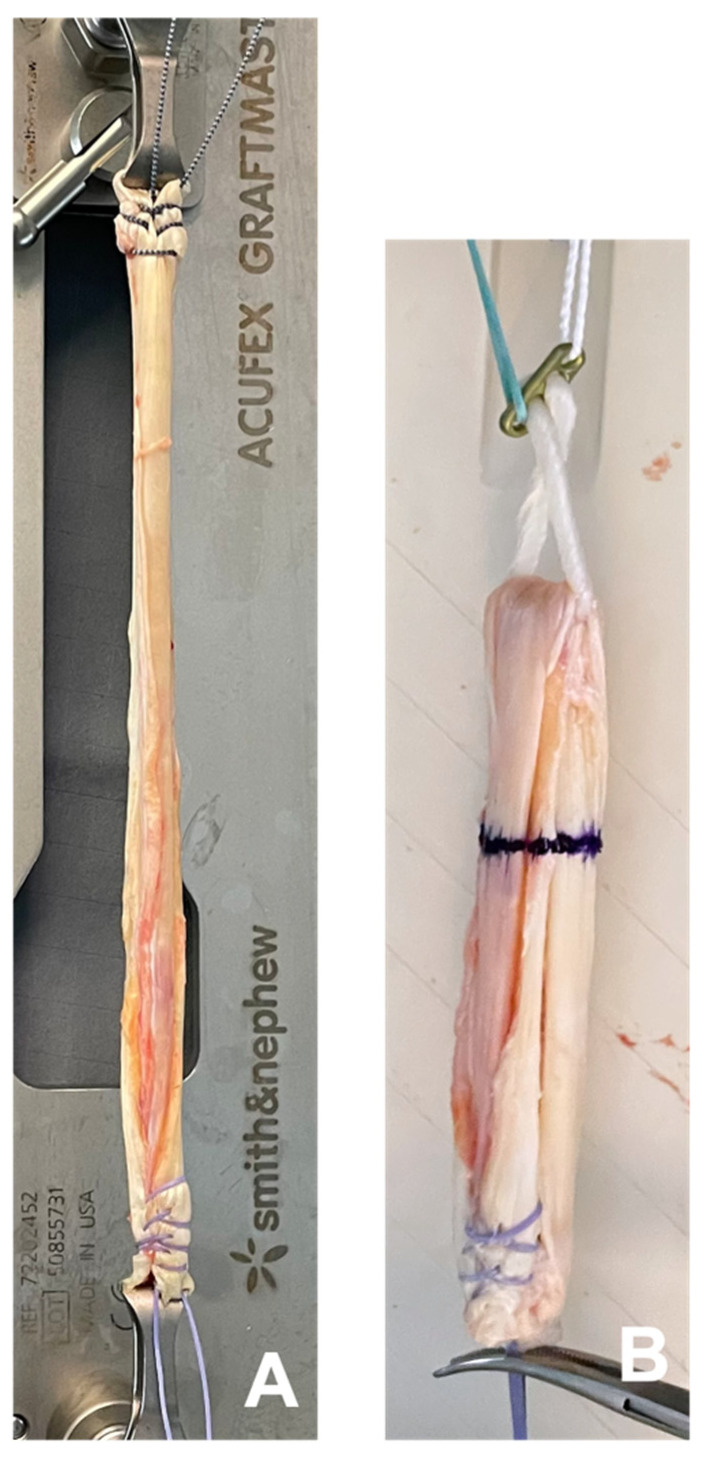
Intraoperative preparation of the RT. (**A**) shows a double-layered rectus tendon graft. The tendon was armored with Orthocord™ Sutures. A 4-fold graft was created using the EndoButton™ CL ultra (**B**).

The technique was previously described in detail [18]. An approximately 3 cm vertical incision was performed just above the upper patellar margin to expose the rectus femoris tendon. A central tendon segment measuring 6–8 mm in width was then carefully mobilized by blunt dissection along its sides. Using an open stripper, the tendon was detached proximally. The knee was then extended, and the tendon was isolated further distally with the scalpel and scissors to the proximal patellar pole. Tendon thickness was adjusted based on tunnel diameter measurements and the desired final graft diameter (targeting 8.5–10 mm). In cases of previous tunnel enlargement, additional tendon substance was included distally to ensure proper graft fit. At this point, the tendon was detached (Figure 1). The tendon underwent debridement of muscle tissue, and a 4-fold graft was formed after reinforcing the tendon ends with Orthocord™ Suture (Depuy Synthes, Raynham, MA, USA) (Figure 2). The graft was presoaked with vancomycin (5 mg/mL) for five minutes before insertion.

### 2.3. Harvest of HT

Through an anteromedial skin incision at the tibia metaphysis, the HT was harvested in a standard manner using a closed stripper. A 4-fold graft was created using the semitendinosus and gracilis tendon. The tendon ends were armored with Orthocord™ Suture (Depuy Synthes, Raynham, MA, USA).

### 2.4. ACL Reconstruction Technique

The graft’s thickness determined the canal’s diameter. The femoral bone socket was accessed through the antero-medial portal. A drill targeting the native ACL footprint was used to create a tibial bone tunnel of the same thickness. The graft was then passed through both the tibial and femoral tunnels, and the EndoButton™ CL ultra (Smith & Nephew, Watford, UK) was flipped in place on the femoral side. With 30 degrees of knee flexion, the graft was fixed on the tibial side with a hybrid fixation using a BioSure™ PK interference screw (Smith & Nethew, Watford, UK) and a bone bridge or a Multifix™ S Ultra (Smith & Nethew, Watford, UK).

### 2.5. Rehabilitation

Without concomitant intraarticular injuries or additional procedures, the post-treatment regimen was as follows: Wearing a prophylactic knee brace with a free range of motion was recommended for about six weeks. Partial weight bearing with crutches was allowed immediately for at least 2 weeks. Following this, full weight bearing was permitted according to the patients’ pain threshold. Physiotherapy sessions were conducted 2–3 times a week for a minimum of 8–12 weeks. Patients were advised to avoid contact sports for 6–9 months based on the state of muscle condition.

### 2.6. Clinical Examination

Data regarding preoperative and postoperative ACL stability grading utilizing the Lachman test were gathered by the surgeon at each follow-up. Mild (grade I) is defined as 0 to 5 mm, moderate (grade II) as 6 to 10 mm, and severe (grade III) as 11 to 15 mm of anterior tibial translation compared to the uninjured side. Additionally, information on the range of motion (ROM) was collected. Intraoperative data included measurements of the autograft’s length and thickness and any associated injuries to the meniscus and collateral ligaments. Furthermore, the type and frequency of postoperative complications and soft tissue irritation, such as pain, swelling, and wound healing disorders, were documented.

### 2.7. Patient-Reported Outcome Measurements (PROMs)

The International Knee Documentation Committee (IKDC) score, Lysholm score, Tegner activity level, and numeric rating scale (NRS) for pain (scores ranging from 0 to 10 points) were completed by the patient at the final follow-up.

### 2.8. Statistical Analysis

Descriptive statistics are presented as mean standard deviation (SD) and range for metric values and as median with interquartile range (IQR) for ordinal variables. The Levene test was used to perform standard distribution testing. For continuous and normal distributed values (IKDC, Lysholm, and ROM), the unpaired *t*-test was used. For ordinal data or non-distributed data (Tegner activity level and NRS), the Mann–Whitney *U* test was used. The calculation regarding stability using the Lachman test in the preoperative and postoperative comparison was carried out using the Wilcoxon test. The chi^2^ test was used to test relationships between categorical variables. All *p*-values were 2-sided with a significance level of *p* ≤ 0.05. No a priori sample size calculation was performed as all eligible patients treated with an RT autograft during the study period were included. The HT group was matched accordingly. This may limit statistical power and is acknowledged as a limitation. Statistical analysis was performed using SPSS v.28.0 (IBM Corp., New York, NY, USA).

## 3. Results

A total of 28 patients (23 males and 5 females) were included in the RT autograft group, and 27 patients (19 males and 8 females) were in the HT autograft group. Patient demographics and the numbers of previous surgeries on the same knee are presented in Table 1. All patients had at least one ACL reconstruction in their patient history.

A two-stage procedure was performed in 10 cases in the HT group and only in 5 cases in the RT group. The two-stage procedure was defined as a revision approach requiring an initial surgery for bone grafting of compromised or widened tunnels, followed by a delayed second-stage ACL reconstruction after sufficient osseous healing. The mean tibial tendon diameter was 9.2 mm (range, 7.5–10 mm) for the RT and 8.5 mm (range, 7.0–10 mm) for the HT. The mean femoral tendon diameter was 9.0 mm (range, 7.5–10 mm) for the RT and 8.2 mm (range 7.0–9.5 mm) for the HT. The surgery time was longer in the RT group (mean 114 vs. 93 min). Notably, more extensive procedures were performed in the RT group (two high tibial osteotomy procedures for medial overload syndrome in genu varum; four lateral extraarticular tenodesis (LET) procedures modified according to Lemaire for additive stabilization) [23]. This could have potentially influenced the results. The number of concomitant injuries to the medial and lateral meniscus and collateral ligaments are presented in Table 2.

### 3.1. Clinical Outcome and Postoperative Complications

Stability, measured using the Lachman test, improved significantly in both groups from a preoperative score of 2 (IQR, 2–3) to 0 (IQR, 0–1) for the RT group and 0 (IQR, 0–2) for the HT group (*p* < 0.001) at the final follow-up (Table 3). In the RT group, three patients showed a persistent extension deficit of approximately 5° compared to one patient in the HT group (*n.s. p* = 0.317). Re-rupture of the ACL graft was observed in two patients in both groups. A total of seven patients underwent revision surgery in the RT group, while four patients underwent revision surgery in the HT group (*n.s. p* = 0.345). In the RT group, two patients were revised because of cyclops syndrome with extensor deficit, two others had persistent meniscal symptoms, two patients received an ACL reconstruction after re-rupture, and one patient underwent a revision surgery due to collision of the femoral endobuttons from previous operations. All of our autografts were placed in a 5 min vancomycin bath before being implanted. We could not document any case of infection. Only a minority of patients in both groups reported donor site pain during the early postoperative phase, which resolved within days. In the HT group, one patient experienced prolonged pain at the contralateral harvesting site for about three months.

### 3.2. Patient-Reported Outcome Measurements

Complete results of the IKDC score, the Lysholm score, and the NRS of pain as well as the Tegner activity level score are presented in Table 3. No significant differences were observed between the groups in any of these scores at the final follow- up. A total of 22 patients (79%) with an RT autograft and 17 patients (63%) with an HT autograft were pain-free (NRS = 0). At the final follow-up, six patients with an RT autograft (21.4%) and three with an HT autograft (11.1%) reached a Tegner activity scale score of 7 or higher.

## 4. Discussion

This study demonstrates that RT and HT autografts provide similar functional outcomes in patient-reported outcome measures (PROMs) for ACL revision surgery. Good tendon quality with parallel fibers and adjustable thickness characterize the RT autograft. A tendency towards a lower rate for a two-stage surgery with the RT was observed.

Due to the excellent postoperative results after primary ACL reconstruction, patients’ athletic demands increase, which in turn raises the risk of retears and the need for associated revisions [2]. Graft selection, graft fixation, and the question of whether a one-stage or two-stage reconstruction with bone grafting should be used remain difficulties in revision ACL surgery [24,25,26]. Due to the average thickness of 9 mm as a 4-fold graft, the use of the RT shows a trend toward reducing the need for a two-stage procedure in cases of bone tunnel enlargement compared to the HT (17.9% vs. 37.0%, not statistically significant). Furthermore, the larger femoral tunnel diameter in the RT group may enhance graft fit and fixation, supporting greater primary stability in revision settings. However, biomechanical studies are needed to confirm this potential advantage.

The risk of retear depends to a certain extent on the diameter of the autograft. Several studies have shown that a lower diameter, especially below 8 mm, is associated with a higher re-rupture rate [6,27,28,29]. HT grafts are thinner than the total QT, the patella tendons, or even the RT as a superficial part of the QT. Our study did not find a higher re-rupture rate, as two patients re-ruptured in each group (7.1% vs. 7.4%).

In the past few years, there has been an increased focus on soft tissue QT autograft in ACL reconstruction. As an alternative to the HT and patellar tendon, QT grafts have shown high scores in PROMs and functional outcomes [7,11,16,30,31,32]. Furthermore, the complication rate and donor site morbidity seem to be lower when using the QT [10,31]. With the use of an RT autograft, we could barely document any complaints at the donor site. Only a minority of patients reported mild pain. However, we noticed an increased rate of postoperative extension deficits and subsequently diagnosed cyclops syndrome (two patients in the RT group). The thickness of the graft and the musculotendinous junction of the RT, which may lead to intraarticular tissue growth, as in cyclops syndrome, might explain this. However, cyclops syndrome was not reported in any of the subsequent studies on the rectus femoris [19,20,21].

In the present study, we compared the PROMs after revision ACL reconstruction with isolated RT and HT autografts. The IKDC score, Lysholm score, Tegner activity levels, and the pain score on the NRS improved. There were no significant differences between the groups. Comparable results between QT and HT autografts have been shown in many studies [5,6,11,16]. Mouarbes et al. [31] showed better outcomes for quadriceps tendon grafts compared to the HT with similar survival rates in a meta-analysis. Lind et al. [16] found no difference between the HT and QT autograft groups. Less donor site morbidity has been documented in the QT group. Barie et al. [5] analyzed the functional outcome in revision ACL reconstruction between the QT and HT. This study also showed comparable functional outcomes. Although our findings align with previous reports on QT grafts, it is essential to note that the isolated RT represents only a portion of the QT. To date, no clinical study directly compares full QT to isolated RT grafts.

Various techniques for harvesting the rectus femoris have been published [19,20,21]. Using our technique, the RT was identified through a skin incision a few centimeters proximal to the patella, where different parts of the QT could be more easily separated. Quyen et al. [19] identified a comparable area proximal to the patella. In contrast, Thamrongskulsiri et al. [21] and Raman et al. [20] harvested the tendon directly from the upper pole of the patella. The size of the skin incision was reported to be between 2 and 3 cm in all studies [19,20,21].

Notably, both techniques for tendon harvesting (HT/RF) can be performed using standard instruments (closed/open stripper) and do not require specialized equipment.

### Strengths and Limitations of This Study

The strengths of this study are the homogenous patient characteristics and the strict inclusion and exclusion criteria. All patients underwent ACL reconstruction using the same technique performed by a single experienced knee surgeon.

This study has several limitations. Muscular strength at the final follow-up was not measured. More detailed biomechanical analyses of the RT autograft as well as further prospective analyses must be performed in order to be able to make more precise statements regarding the clinical outcome and postoperative quadriceps muscle strength. It should be noted that several patients underwent additional procedures, such as high tibial osteotomy or lateral extraarticular tenodesis. These interventions could have affected rehabilitation progress and influenced the PROMs. Other limitations are the small sample size and the lack of randomization, which both bias the results and make it difficult to identify possible uncommon complications. Another area for improvement is the lack of objective stability measurements, such as the KT-1000 arthrometer or pivot-shift grading. Stability was evaluated solely via the Lachman test by the operating surgeon, which may introduce bias and does not fully capture rotational stability. The lack of quantitative measurement of donor site morbidity, such as strength testing, represents a limitation of this study as it could have provided additional valuable insights into the functional outcomes of graft alternatives.

## 5. Conclusions

RT and HT autografts show comparable scores in PROMs. There is a tendency toward fewer two-stage procedures using the RT instead of the HT. Especially in revision settings with tunnel widening or when HT autografts are not available, the isolated RT provides a technically feasible and effective alternative.

## Figures and Tables

**Table 1:** Patients’ characteristics.

		RT	HT
N total		28	27
Male (%)		82%	70%
Age (year)		29.7 (18.7–51.7)	32.3 (12.8–53.4)
BMI (kg/m^2^)		25.6 (20.8–37.2)	25.1 (18.0–39.3)
Follow-up (month)		40.3 (16.4–64.8)	61.2 (34.6–86.3)
Previous surgeries (N)	1	17	13
	2	9	12
	3	2	2

*RT*, Rectus Tendon Autograft; *HT*, Hamstring Tendon Autograft; N, Number; *BMI*, Body Mass Index. Numeric parameters: mean (range).

**Table 2:** Intraoperative measurements.

	RT	HT	*p*-Value
Bore canal filling preop. (%)	17.9	37.0	n.s. (0.11) ^a^
Medial meniscus injury (%)	64.3	66.7	n.s. (0.85) ^a^
Lateral meniscus injury (%)	53.6	22.2	n.s. (0.17) ^a^
Collateral ligament injury (%)	10.7	0	n.s. (0.08) ^a^
Time of surgery (minute)	114 (68–182)	93 (34–214)	0.021 ^b^
Femoral tendon thickness (mm)	9.0 (7.5–10.0)	8.2 (7.0–9.5)	<0.001 ^b^
Tibial tendon thickness (mm)	9.2 (8–10.0)	8.5 (7.0–10.0)	<0.001 ^b^

*RT*, Rectus Tendon Autograft; *HT*, Hamstring Tendon Autograft; *Preop*., preoperative; *mm*, millimeter. Numeric parameters: mean (range). ^a^ Chi^2^ test; ^b^ unpaired *t*-test.

**Table 3:** Outcomes and PROMs of ACL reconstruction with RT and HT autografts.

		Rectus (n = 28)	Hamstrings (n = 27)	*p*-Value
Lachman Test	Preoperative Period Final Follow-Up *p*-value	2 (Q1 = 2; Q3 = 3) 0 (Q1 = 0; Q3 = 0) <0.001 ^b^	2 (Q1 = 2; Q3 = 3) 0 (Q1 = 0; Q3 = 1) <0.001 ^b^	n.s. (0.87) ^a^ n.s. (0.22) ^a^
IKDC Score	Final Follow-Up	74.7 ± 10.9	74.9 ± 12.9	n.s. (0.96) ^c^
Lysholm Score	Final Follow-Up	90.9 ± 15.0	89.0 ± 14.6	n.s. (0.62) ^c^
Tegner Activity	Final Follow-Up	5 (Q1 = 4; Q3 = 6)	5 (Q1 = 4; Q3 = 6)	n.s. (0.66) ^a^
NRS	Final Follow-Up	0 (Q1 = 0; Q3 = 0)	0 (Q1 = 0; Q3 = 1)	n.s. (0.29) ^a^
Re-Rupture		2 (7.1%)	2 (7.4%)	n.s. (0.97) ^d^

*RT*, Rectus Tendon Autograft; *HT*, Hamstring Tendon Autograft; *IKDC*, International Knee Documentation Committee score; *NRS*, Numerical Rating Scale. ^a^ Mann–Whitney U test; ^b^ Wilcoxon test; ^c^ unpaired *t*-test; ^d^ chi^2^ test.

## Data Availability

The original data presented in this study are openly available in FigShare at https://doi.org/10.6084/m9.figshare.28794023.v1 (accessed on 15 April 2025).
